# Bisphosphonate-Conjugated Sitafloxacin for Treatment of *Staphylococcus aureus* Infection Associated with Cortical Bone Screws: Case Series in Sheep Model

**DOI:** 10.3390/ph18050675

**Published:** 2025-05-01

**Authors:** Niels Vanvelk, James Tapia-Dean, Stephan Zeiter, Karen de Mesy Bentley, Chao Xie, Frank Hal Ebetino, Shuting Sun, Jeffrey Neighbors, Edward M. Schwarz, Thomas Fintan Moriarty

**Affiliations:** 1 AO Research Institute Davos, 7270 Davos, Switzerland; 2 Trauma Research Unit, Department of Surgery, Erasmus MC, University Medical Center, 3015GD Rotterdam, The Netherlands; 3 Center for Musculoskeletal Research, University of Rochester Medical Center, Rochester, NY 14642, USA; 4 Center for Advanced Research Technology (CART), Electron Microscopy Resource, University of Rochester Medical Center, Rochester, NY 14642, USA; 5 Department of Pathology and Laboratory Medicine, University of Rochester Medical Center, Rochester, NY 14642, USA; 6 Department of Chemistry, University of Rochester, Rochester, NY 14642, USA; 7 BioVinc LLC, Pasadena, CA 91107, USA; 8 Department of Chemistry, University of Southern California, Los Angeles, CA 90089, USA; 9 Department of Pharmacology, Pennsylvania State University College of Medicine, Hershey, PA 17033, USA; 10 Center for Musculoskeletal Infections (ZMSI), University Hospital Basel, 4031 Basel, Switzerland

**Keywords:** implant-associated bone infection, methicillin-resistant *Staphylococcus aureus* (MRSA), sheep model, bisphosphonate-conjugated antibiotics (BCAs), osteocyte lacuno-canalicular network

## Abstract

**Background/Objectives**: Hydroxybisphosphonate-conjugated sitafloxacin (HBCS) was developed to achieve higher antibiotic concentrations within infected bone. Small animal studies supported further development, but the feasibility of HBCS treatment in a more clinically relevant and larger animal model is unknown. **Methods**: In this study, we present case reports on four sheep, each receiving four MRSA-contaminated tibial screws treated with different regimens of intravenous antibiotics. The first two sheep received two screws contaminated with 10^3^ CFU and two screws contaminated with 10^5^ CFU. Sheep 1 only received vancomycin, starting on day two. Sheep 2 received vancomycin, starting on day 2, but also received 7 doses of HBCS (2 mg/kg/48 h). The protocol for the final two sheep was revised, and both received four screws contaminated with 10^3^ CFU, and vancomycin was started preoperatively. Sheep 3 and 4 received 7 doses (starting on day 6) and 9 doses (starting on day 2) of HBCS (4 mg/kg/48 h), respectively. Bacteriology was performed on three screws per animal. Longitudinal radiography and histology (n = 1 screw) were assessed for signs of osteolysis and reactive bone formation. Electron microscopy (EM) was performed in the first two sheep to evaluate antibiotic-induced bacterial damage. **Results**: All sheep tolerated HBCS infusion without clinical signs of discomfort. In addition to a high bacterial load (~10^4^ CFU on all screws), Sheep 1 displayed extensive radiographic and histologic evidence of peri-implant osteolysis and reactive bone formation. Despite having a high bacterial load (~10^4^ CFU on all screws), Sheep 2 displayed only mild radiographic and histologic evidence of peri-implant osteolysis and periosteal reactive bone formation. Bacteriology in Sheep 3 and 4 demonstrated near MRSA eradication (<100 CFU on 2 screws). Both sheep displayed no evidence of osteolysis or new bone formation adjacent to the screw head. EM confirmed the presence of bacteria resorbing bone and replicating in biofilm in Sheep 1, while antibiotic-killed bacteria with ruptured septal planes were seen in Sheep 2. **Conclusions**: This study demonstrates the feasibility of HBCS therapy in a clinically relevant animal model and provides guidance on future efficacy studies, such as the use of an inoculum of 10^3^ CFU per screw, the initiation of antibiotic treatment commencing at the time of surgery, and the usability of antibiotic-killed bacteria within altered glycocalyx observed by TEM as a potential biomarker for HBCS efficacy.

## 1. Introduction

Musculoskeletal infection (MSKI) comprises a collection of clinical entities, including fracture-related infection (FRI), prosthetic joint infection (PJI), septic arthritis, and diabetic foot osteomyelitis [1,2,3]. Despite recent advances towards standardized prevention and treatment protocols, MSKI still places a heavy burden on both the patient and healthcare system. The costs associated with infection have been reported to be up to 6.5 times higher compared to non-infected cases [4]. This can be explained by a high recurrence rate, leading to frequent revision surgeries and prolonged antibiotic therapy [5]. As the primary pathogen responsible for bone infection, *Staphylococcus aureus* possesses multiple pathogenic mechanisms enabling it to invade and survive within bone [2]. One mechanism is the invasion and colonization of the osteocyte lacuno-canalicular network (OLCN). This was first demonstrated in an in vivo model of chronically infected mouse bones and later in a case report of diabetic foot osteomyelitis in a human patient [6,7]. A subsequent study identified Penicillin Binding Protein 4 (PBP4) as a crucial factor for OLCN invasion and demonstrated a higher efficacy of standard-of-care (SOC) antibiotics against ΔPBP4-mutated strains [8].

To overcome the biodistribution limits of SOC antibiotics, bisphosphonate-conjugated antibiotics (BCAs) were developed [9]. Bisphosphonates are a derivate of inorganic pyrophosphate (PPi), which is composed of two phosphate groups linked by a metabolically stable carbon atom. Bisphosphonates have a high affinity for hydroxyapatite, particularly at high turnover sites of the skeleton where there are free surface areas due to active bone metabolism. This affinity for bone allows BCA to be delivered specifically to the bone–bacteria interface, where bone resorption takes place. We have identified three BCAs not related to our previous work. In their first study, Sedghizadeh et al. showed a high-binding of ciprofloxacin-conjugated bisphosphonate to hydroxyapatite and its efficacy against methicillin-sensitive *S. aureus* (MSSA), methicillin-resistant *S. aureus* (MRSA), *P. aeruginosa*, and *A. actinomycetemcomitans* in vitro and in vivo in a rat model of implant-associated osteomyelitis [10]. In a follow up study, these investigators evaluated quinolone antibiotics and several bisphosphonate-conjugated versions of these antibiotics and found that they were able to inhibit *S. aureus* biofilms in a dose-dependent manner [11]. Adjei-Sowah et al. demonstrated the cytolytic activities of bisphosphonate and hydroxybisphosphonate conjugates of sitafloxacin and tedizolid against MSSA and MRSA on bone wafers in vitro [9]. With appropriate linker selection, the antibiotic can be released at the infection site following acid and/or enzymatic cleavage [9]. Importantly, the BCA strategy described here utilizes non-nitrogen-containing bisphosphonates that possess a high affinity to hydroxyapatite at resorbing bone surfaces while having negligible biological effects on bone cells [12,13].

To select the ideal partner antibiotic within the BCA, a large screen was performed of available FDA-approved drugs [14]. Sitafloxacin was found to be the most effective bactericidal drug against both methicillin-sensitive *S. aureus* (MSSA) and methicillin-resistant *S. aureus* (MRSA) in static biofilms in vitro [14] and in preclinical models of osteomyelitis [9,11,15,16]. In addition, sitafloxacin has a broad antibiotic spectrum of activity [17]. Hydroxybisphosphonate-conjugated sitafloxacin (HBCS) was consequently designed and synthesized by linking sitafloxacin to 1-hydroxy-2-(4-hydroxyphenyl)ethane-1,1-diyl)bis(phosphonic acid) via a semi-stable phenyl carbamate component [9]. The ability of HBCS to kill *S. aureus* has thereafter been demonstrated in both in vitro experiments and murine models of implant-associated infections, confirming significant antibacterial activity against bacteria on mineral surfaces [9,15,18]. After treatment with BCA, electron microscopy (EM) revealed bacteria containing small vacuoles and holes [15,18], which is consistent with antibiotic-induced vacuolization, bacterial swelling, and a necrotic phenotype [19,20]. ln addition, tartrate-resistant acid phosphatase (TRAP)-staining demonstrated that HBCS significantly reduces the number of peri-implant osteoclasts compared to placebo and free sitafloxacin controls [15,18]. These results support the “target and release” strategy of BCA to overcome the biodistribution limits of SOC antibiotics in mice. However, the feasibility of BCA therapy in a more clinically relevant animal model is still unknown. Thus, with this study, we aimed to develop a sheep model representing the clinical situation of an orthopedic device-related infection (ODRI) caused by an inoculum of MRSA that is sufficient to reproducibly establish non-life-threatening implant-associated bone infections. This model was subsequently utilized to evaluate the tolerability and feasibility of HBCS therapy in this clinically relevant scenario and to collect pilot safety and efficacy data to instruct the design of future large animal studies.

## 2. Results

### 2.1. Animal Welfare

No sheep were excluded from this study, and all sheep survived the entire predefined study duration. The treatment for each sheep was adjusted based on the experience and insights gained from the preceding sheep. In Sheep 1 and 2, swelling and redness at the surgical site were noted for the entire study duration. Increasing signs of pain necessitated the administration of supplementary analgesia. Subsequently, for Sheep 3 and 4, vancomycin therapy was initiated prophylactically, and only screws with a low inoculum were inserted. Additionally, the screw length was shortened for Sheep 3 and 4, resulting in less protrusion of screw ends into soft tissues. The severity of local infection signs decreased after the start of antibiotic administration in Sheep 1 and 2. Only Sheep 3 lost bodyweight (1 kg) over the study duration.

### 2.2. Sheep One

**Figure 1:**
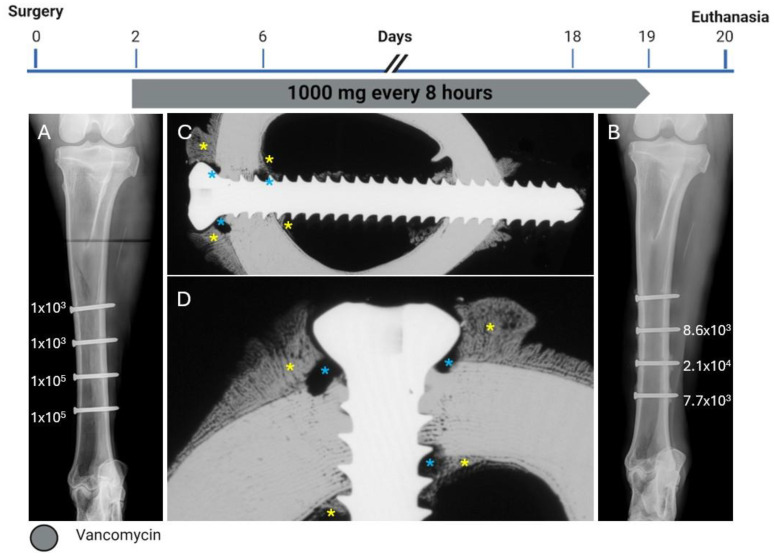
Treatment timeline, radiographs, and bacteriology of Sheep 1. (**A**) anteroposterior radiograph immediately postoperative showing the corresponding inoculation CFU count per screw next to each screw. (**B**) Anteroposterior radiograph after euthanasia showing the corresponding persistent CFU count per screw at the end of the study next to each screw. (**C**,**D**) Contact radiograph of a transverse section of the proximal screw after euthanasia at different magnifications showing extensive osteolysis (blue asterisks) and periosteal and endosteal reaction (yellow asterisks) next to the screw.

**Figure 2:**
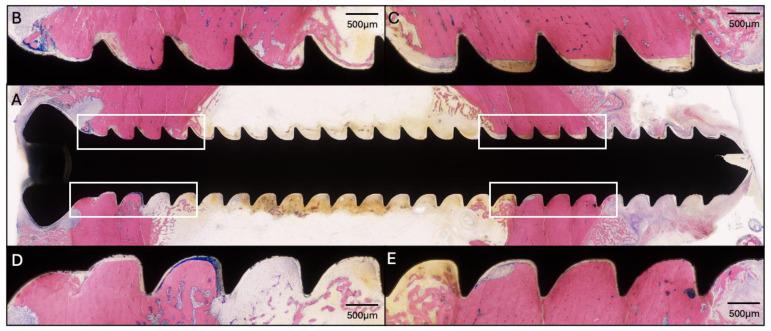
Histology of the proximal screw of Sheep 1 using Giemsa and Eosin staining. (**A**) Overview image at 4× magnification (center). (**B**–**E**) Magnified images of the corresponding areas marked in (**A**) at 20× magnification showing osteolysis around the screw threads, primarily at the head of the screw.

This sheep received all four screws with a length of 34 mm. The radiographic and microbiology results for Sheep 1 that did not receive any HBCS treatment are presented in Figure 1. At the end of the study, the CFU for the low inoculum screw was 8.6 × 10^3^. The average CFU for the two high inoculum screws was 1.4 × 10^4^ (range 7.7 × 10^3^–2.1 × 10^4^). The removal torque for the low inoculum screw was 1.1 Nm. The average removal torque for the two high inoculum screws was 0.9 Nm. WBC peaked at 7.6 × 10^9^/L (day 3) and decreased thereafter. G&E staining of the proximal screw is displayed in Figure 2. Radiographic and histological observations were consistent, with areas of osteolysis observed around the screw head, with extensive adjacent periosteal reaction.

### 2.3. Sheep Two

**Figure 3:**
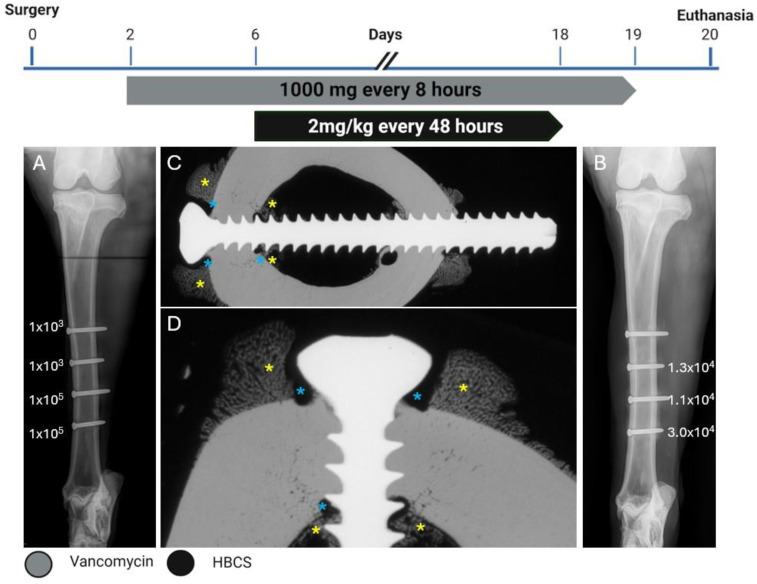
Treatment timeline, radiographs, and bacteriology of Sheep 2. (**A**) Anteroposterior radiograph immediately postoperative showing the corresponding inoculation CFU count per screw next to each screw. (**B**) Anteroposterior radiograph after euthanasia showing the corresponding persistent CFU count per screw at the end of the study next to each screw. (**C**,**D**) Contact radiograph of a transverse section of the proximal screw after euthanasia at different magnifications showing mild osteolysis (blue asterisks) and periosteal and endosteal reaction (yellow asterisks) next to the screw.

**Figure 4:**
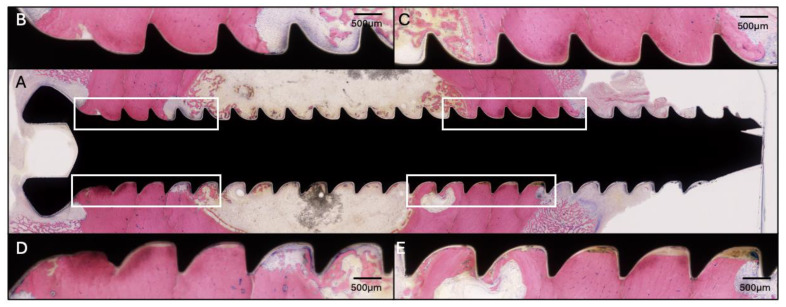
Histology of the proximal screw of Sheep 2 using Giemsa and Eosin staining. (**A**) Overview image at 4× magnification. (**B**–**E**) Magnified images of the corresponding areas marked on (**A**) at 20× magnification showing little osteolysis around the screw threads.

The screw lengths for this sheep were 34, 30, 34, and 30 mm from proximal to distal. Sheep 2 started HBCS treatment on day 6 and received 7 administrations of 2 mg/kg (Figure 3) in addition to the vancomycin treatment starting on the second day after surgery. No adverse clinical signs were detected upon infusion. At the end of the study, the CFU for the low inoculum screw was 1.3 × 10^4^. The average CFU for the two high inoculum screws was 2.1 × 10^4^ (range 1.1 × 10^4^–3.0 × 10^4^). The removal torque for the low inoculum screw was 1.1 Nm. The average removal torque for the two high inoculum screws was 1.2 Nm. WBC peaked at 9.7 × 10^9^/L (day 3) and decreased thereafter. G&E staining of the proximal screw is displayed in Figure 4. Radiographic and histological observations were consistent, with areas of mild osteolysis observed, once again, around the screw head, with adjacent periosteal and endosteal reaction.

### 2.4. Sheep Three

**Figure 5:**
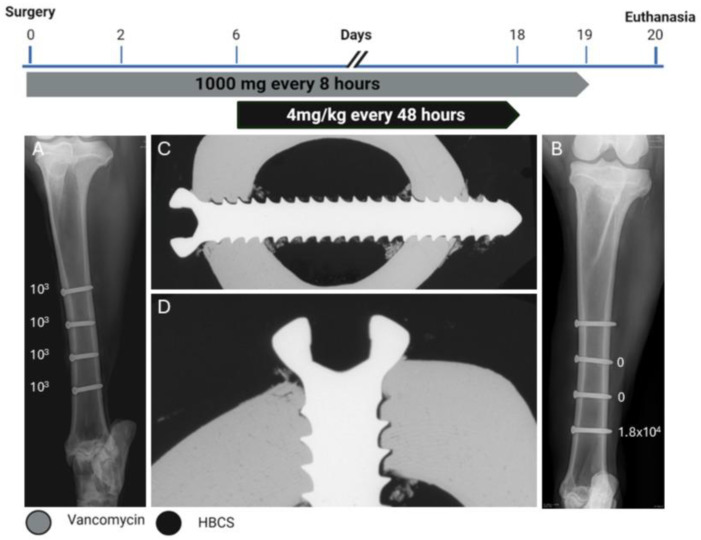
Treatment timeline, radiographs, and bacteriology of Sheep 3. (**A**) Anteroposterior radiograph immediately postoperative showing the corresponding inoculation CFU count per screw next to each screw. (**B**) Anteroposterior radiograph after euthanasia showing the corresponding persistent CFU count per screw at the end of the study next to each screw. (**C**,**D**) Contact radiograph of a transverse section of the proximal screw after euthanasia at different magnifications showing no osteolysis and periosteal and endosteal reaction next to the screw.

**Figure 6:**
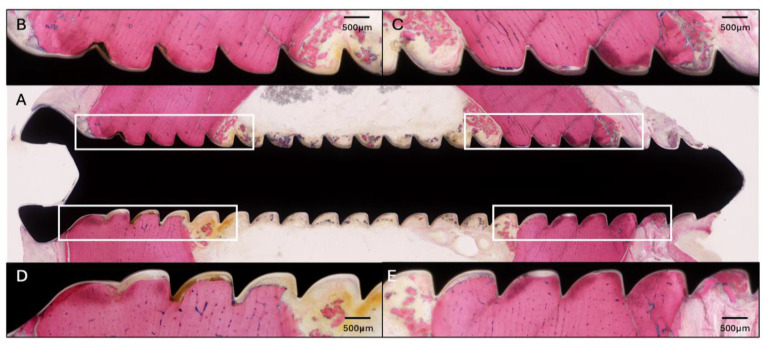
Histology of the proximal screw of Sheep 3 using Giemsa and Eosin staining. (**A**) Overview image at 4× magnification. (**B**–**E**) Magnified images of the corresponding areas marked on (**A**) at 20× magnification showing no osteolysis or periosteal reaction around the screw head.

Intraoperatively, screws of the following length were inserted from proximal to distal: 28, 26, 26, and 28 mm. Sheep 3 started HBCS treatment on day 6 and received 7 administrations of 4 mg/kg (Figure 5). At the end of the study, the average CFU for the three low inoculum screws was 5.8 × 10^3^ (range 0–1.75 × 10^4^). The average removal torque for the three screws was 1.3 Nm. WBC peaked at 4.4 × 10^9^/L (day 15) and decreased thereafter. G&E staining of the proximal screw is displayed in Figure 6. Radiographic and histological observations were, once again, consistent, although osteolysis was not observed around the screw head, and there was no periosteal reaction.

### 2.5. Sheep Four—HBCS 4 mg/kg—9 Administrations

**Figure 7:**
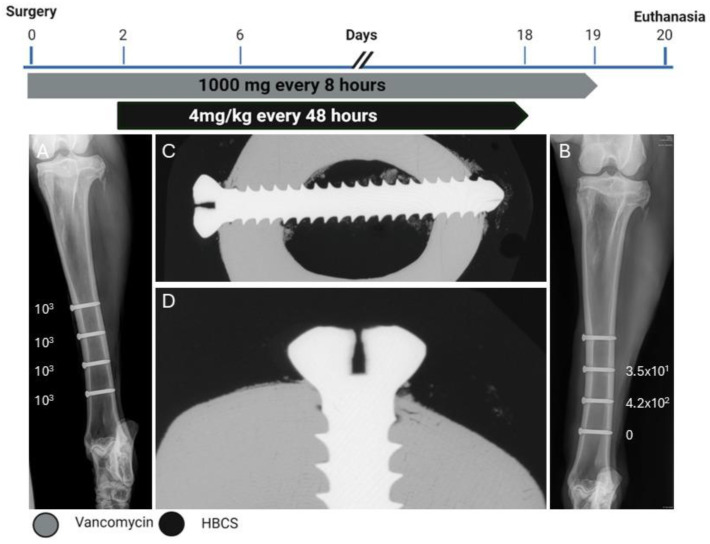
Treatment timeline, radiographs, and bacteriology of Sheep 4. (**A**) Anteroposterior radiograph immediately postoperative showing the corresponding inoculation CFU count per screw next to each screw. (**B**) Anteroposterior radiograph after euthanasia showing the corresponding persistent CFU count per screw at the end of the study next to each screw. (**C**,**D**) Contact radiograph of a transverse section of the proximal screw after euthanasia at different magnifications showing no osteolysis and periosteal reaction next to the screw head.

**Figure 8:**
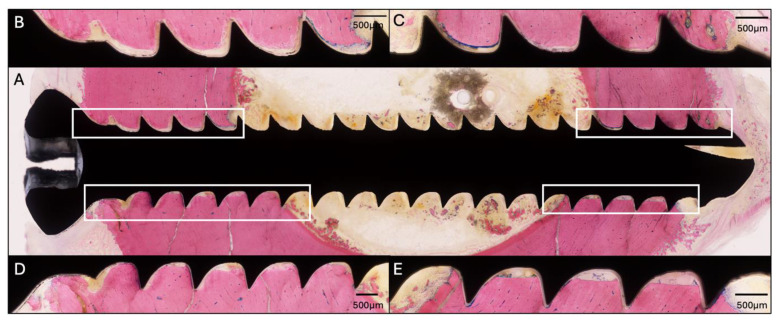
Histology of the proximal screw of Sheep 4 using Giemsa and Eosin staining. (**A**) Overview image at 4× magnification. (**B**–**E**) Magnified images of the corresponding areas marked on (**A**) at 20× magnification showing no osteolysis or periosteal reaction around the screw head.

The screw lengths inserted were 30, 28, 28, and 30 mm (from proximal to distal). Sheep 4 started HBCS treatment on day 2 and received 9 administrations of 4 mg/kg (Figure 7). At the end of the study, the average CFU for the three low inoculum screws was 1.5 × 10^2^ (range 0–4.2 × 10^4^). The average removal torque for the three screws was 1.2 Nm. WBC peaked at 6.6 × 10^9^/L (day 3) and decreased thereafter. G&E staining of the proximal screw is displayed in Figure 8. Radiographic and histological observations were once again consistent and similar to Sheep 3. Osteolysis was not observed around the screw head, and no periosteal reaction was observed.

### 2.6. Transmission Electron Microscopy

**Figure 9:**
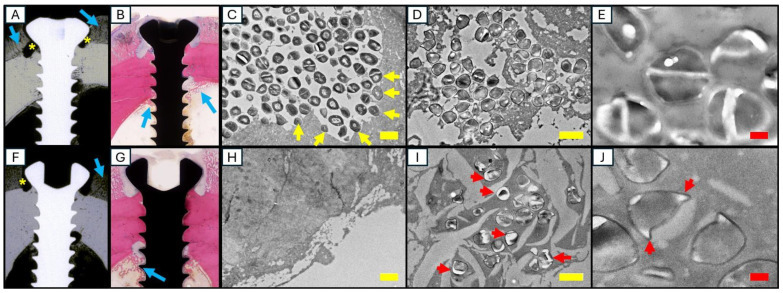
Radiology, histology, and TEM analyses of Sheep 1 and 2. A lack of vancomycin monotherapy efficacy in Sheep 1 was evident from the peri-implant osteolysis (yellow asterisk) and the reactive bone formation on all cortices that flank the screw (blue arrows) in the X-ray (**A**) and Giemsa-stained histology (**B**). As confirmation, TEM analysis revealed large numbers of bacteria actively resorbing the cortical bone (yellow arrows in (**C**)) and replicating bacteria in biofilm (**D**,**E**). In contrast, these pathologies were markedly reduced in Sheep 2 receiving vancomycin and 7 infusions of 2 mg/kg of HBCS. This reduced peri-implant osteolysis and reactive bone formation that flanked the screw (**F**,**G**). Moreover, no bacteria were present on the bone of HBCS-treated sheep (**H**), and most of the bacteria in the peri-implant biofilm had features of cell death and ruptured septal planes consistent with antibiotic killing (red arrow in (**I**,**J**)). Yellow scale bars indicate 1 µm, red scale bars indicate 200 nm.

TEM of cortical bone fragments recovered from the screws of Sheep 1 revealed large numbers of bacteria actively resorbing the bone (Figure 9A–E). TEM of debridement tissue from the screw holes readily identified live bacteria within biofilm. TEM of Sheep 2 showed no bacteria on the bone, and most of the bacteria in the peri-implant biofilm had features of death from antibiotics (Figure 9F–J). Due to the low bacterial burden on the three distal screws and absence of osteolysis and reactive bone formation on the proximal screw, TEM analysis was not performed on screws from Sheep 3 or 4.

## 3. Discussion

Colonization of the OLCN by bacteria, including *S. aureus* [6], hampers the treatment of chronic bone infection by SOC antibiotics. HBCS is being developed as a first-in-class bone-targeting antibiotic to address this major unmet clinical need. Recent studies with murine models of MRSA implant-associated osteomyelitis have demonstrated HBCS to be effective at reducing the bacterial burden on the implants and in tissues, eradicating *Staphylococcus* abscess communities (SACs), preventing peri-implant osteolysis, and facilitating osseous integration of MRSA contaminated implants [15,18]. TEM imaging demonstrated defined phenotypes of vital, autolyzed, and antibiotics killed MRSA in SAC and the OLCN [15,18].

Based on this preclinical success, we aimed to develop a sheep model of an MRSA-induced ODRI that could serve to evaluate the efficacy of HBCS in this clinically relevant scenario and allow for testing different dosing regimens as an adjuvant therapy to SOC antibiotics in future studies. As a first step, several techniques (drug preparation and infusion, surgical implantation of the screws, processing of the tissue and screws postmortem) and variables (inoculum, initiation of antibiotic therapy, and HBCS dose) needed to be explored prior to these formal prospective studies. In the present study, we describe case reports on four sheep, each receiving four bicortical screws, pre-inoculated with different inocula of MRSA. Sheep were consequently treated with different antibiotic regimens. Evaluations of the therapeutic effect were performed by bacteriology, longitudinal radiography, and histology. Although these data cannot be used for statistical analyses and scientific conclusions, there were several remarkable observations that will guide the development of prospective studies with this novel ovine model. First, we did not see remarkable differences in the implant-associated infections between the 10^3^ and 10^5^ CFU inocula used in Sheep 1 and 2. Thus, for animal welfare reasons, we used the 10^3^ CFU inoculum in Sheep 3 and 4, which performed well. Second, shorter screws should be used to avoid tissue damage and micromotion at the distal cortex. Third, antibiotic therapy with HBCS should commence promptly after infection, which is consistent with murine studies demonstrating that the race for the surface of the implant is complete within three h [21] and that mature biofilm formation is complete within 24 h [22]. Fourth, screw removal torque measurements are not justified at this early 20-day time point. We did not see any differences between the screws with high bacterial counts and peri-implant osteolysis to those without signs of infection.

While statistical analysis of the data in this study is not possible, these case reports also support further clinical development of HBCS for implant-associated bone infection. First, we did not experience any challenges with its co-administration with vancomycin, and no complications or adverse events were observed in any of the sheep. Second, sheep treated with high doses of HBCS showed less signs of fulminant infection. Bacteriology showed near eradiation of the bacteria. Radiography and histology showed less osteolysis and reactive bone formation. Third, the TEM results are suggestive of in vivo eradication of infection by HBCS. In the first sheep, bacteria were actively resorbing cortical bone and dividing in biofilm adjacent to the screw. In the second sheep, bacteria were absent from the bone, and bacteria with antibiotic-killed features were visible. By using TEM to differentiate phenotypes of vital, autolyzed, and antibiotic-killed bacteria, earlier studies provided initial evidence that systemic HBCS can kill MRSA in SAC and OLCN [15,18]. These data also support further development of TEM as a biomarker of antibiotic-killed bacteria. This is critical, as the broad field of antimicrobial research is limited by the exclusive use of CFU assays to assess antibiotic efficacy, and a reduction in CFUs could be due to several cytostatic and cytotoxic mechanisms. Thus, academia, industry, and the FDA are actively investigating novel biomarkers of the antibiotic killing of bacteria in vitro, ex vivo, and in vivo.

## 4. Materials and Methods

### 4.1. Study Overview

This study was performed in a staggered approach involving sheep (hereafter named Sheep 1–4 based on time of enrollment in the study). All sheep received four bicortical screws in the right tibia without creating instability (i.e., no fracture or osteotomy). Prior to insertion, the screws were pre-colonized with *S. aureus* USA300. Antibacterial treatment was initiated on the second day after surgery (Sheep 1 and 2) or 1 h prior to surgery (Sheep 3 and 4). Sheep 1 was treated with intravenous vancomycin only. The other sheep received a combination of vancomycin and varying doses of HBCS.

### 4.2. Animals, Ethical Approval, Preoperative Animal Care, Anesthesia, and Analgesia

Four healthy female White Alpine Sheep, aged 3–6 years, were included in this study after being clinically assessed and checked for orthopedic diseases. The sheep were sourced from a flock of Swiss Alpine sheep raised by a local farmer (Urban Lanker, Davos, Switzerland). The sheep were randomly allocated to one of the four study groups. Approval to perform this study was granted by the ethical committee of the canton of Grisons in Switzerland (approval number: 23/2022). All procedures were performed in an Association for Assessment and Accreditation of Laboratory Animal Care (AAALAC) International approved facility. The sheep were group-housed for at least 2 weeks prior to surgery to habituate to housing conditions and were fed twice per day with hay, maize, and salt.

Prior to surgery, each sheep fasted for 24 h and was sedated with an intramuscular (IM) injection of 0.2 mg of detomidine (Equisedan^®^, Graeub AG, Bern, Switzerland) approximately 20 min before transfer to the preparation area. Induction was performed by intravenous (IV) infusion of 0.2 mg/kg of diazepam (Midazolam Sintetica^®^, Sintetica AG, Mendrisio, Switzerland) and 4 mg/kg of ketamine (Ketasol-100^®^, Roche Pharma AG, Grenzach-Wyhlen, Germany). After endotracheal intubation, anesthesia was maintained using sevoflurane, 1.5–3% *v*/*v* in 0.6 L/min of oxygen (Sevoflurane Baxter^®^, Baxter AG, Glattpark, Switzerland). For intraoperative analgesia, the sheep received an epidural injection between sacrum and last lumbar vertebrae with 0.05 mg/kg buprenorphine (Bupaq^®^, Streuli Pharma AG, Uznach, Switzerland) mixed with 2 mg/kg of lidocaine 2% (Lidocain^®^ 2%, Streuli Pharma AG, Uznach, Switzerland) and an injection of 4 mg/kg of carprofen (Cardrodolor^®^ Rind, Pfizer AG, Zürich, Switzerland) IV. Postoperative analgesia included a subcutaneous injection of 4 mg/kg of carprofen every 24–48 h for 5 days, 0.6 mg of buprenorphine (Bupaq^®^, Streuli Pharma AG, Uznach, Switzerland) IM was given directly postoperative and 6 h thereafter, and 2 μg/kg/h of fentanyl (Fentanyl Mepha Matrix patches^®^, Mepha Pharma AG, Aesch, Switzerland) transdermal for 72 h. Supplementary analgesia was given for animals in the postoperative period with increased signs of pain, including Fentanyl, buprenorphine, and Caprofen. Postoperatively, Sheep 1 and 2 were kept in supportive suspension slings for the entire study, which allowed for full weight bearing; however, peak forces were avoided on the leg when standing up. Sheep 3 and 4 were supported for 5 days post-surgery, after which they showed improved weight bearing on the operated leg compared to the previous sheep.

Sheep were always housed in pairs during the entire postoperative period. Animal welfare was checked by clinical examination and documented in the scoresheet for each individual animal. These assessments were performed twice daily during the first week postoperatively by a veterinarian or animal caretaker and then twice weekly. The following parameters were documented to assess the wellbeing of the sheep: general appearance, breathing, food and water uptake, weight, weight bearing of operated limb, rectal temperature, wound healing, and feces. Abruption criteria were formulated based on the scoring of the aforementioned clinical assessment. This study adhered to the ARRIVE reporting standards for research (see ARRIVE guidelines).

### 4.3. Screw Inoculation

An overnight bacterial culture was established by inoculation of MRSA USA300 in 20 mL of tryptic soy broth (TSB), which was incubated overnight at 37 °C. A subculture was subsequently made into sterile TSB and incubated for another 2 h at 37 °C. Consequently, the subculture was centrifugated twice at 3214 relative centrifugal force for 7 min at 20 °C. After each centrifugation, the pellet was resuspended in 20 mL of phosphate-buffered saline (PBS). Next, the suspension was sonicated (Ultrasonic Waterbath RK 510 H, Bandelin operating at 640 W) for 3 min. The optical density was measured (Multiskan GO, Thermo Scientific, Waltham, MA, USA) at 600 nm (OD_600_) and diluted to achieve final inocula (high or low). Screws were incubated in these inocula for 10 min, before being allowed to air dry for another 10 min. The bacterial load on the screw was 10^3^ and 10^5^ CFU/screw for the low and high inocula, respectively, as revealed by a quantitative culture after sonication of the test screws.

### 4.4. Surgical Protocol

After aseptical preparation of the surgical field, a longitudinal skin incision of approximately 10 cm was made on the medial aspect of the distal right tibia, and blunt dissection was performed to expose the medial aspect of the bone. Starting 5 cm above the medial malleolus, 4 bicortical holes were drilled 25 mm apart under constant irrigation with saline. The appropriate screw length was chosen from the pre-inoculated screw lengths available (either a 26, 28, 30, or 34 mm or 4 mm Cortical screw). The screws were inserted so that the screwhead became flush with the cortex without causing overtightening, and the insertion torque was measured using a digital torque measuring tool (Mecmesin, Advanced Force and torque indicator, AFTI). For Sheep 3 and 4, the insertion torque was standardized to approximately 2 Nm. After screw placement, the incision was closed in three layers (fascia, subcutaneous, and skin) with sutures (3–0 noncutting and cutting Monocryl, Ethicon).

### 4.5. Antibiotic Treatment

Vancomycin (Labatec Pharma SA, Geneva Switzerland) was administered intravenously at a regular dose of 1 g every 8 h to all sheep. For Sheep 1 and 2, IV vancomycin was initiated at day 2 postoperatively and continued until euthanasia on day 20. However, by day 2 post-operative, both these sheep displayed a fulminant infection with increased signs of severe pain and discomfort above the expected range, posing an elevated risk of wound dehiscence. To reduce infection burden, Sheep 3 and 4 received vancomycin prophylactically 1 h before surgery, which was continued immediately postoperatively, also at a regular dose of 1 g every 8 h.

### 4.6. HBCS Administration

HBCS (BioVinc, Pasadena, CA, USA) was prepared in sterile milli-Q water, according to the manufacturer’s instructions, and diluted in saline prior to administration. This HBCS was administered intravenously every 48 h for all sheep other than the vancomycin-only control sheep. Sheep 2 received 7 HBCS administrations, starting from day 6 postoperatively at a dose of 2 mg/kg. Sheep 3 and 4 received 7 and 9 administrations of HBCS at a dose of 4 mg/kg, starting from day 6 and 2, respectively. For all sheep receiving HBCS, the last administration was performed 48 h before euthanasia.

### 4.7. In Vivo Analysis and Euthanasia

Bodyweight was measured for each sheep preoperatively and weekly thereafter. Radiographs were made prior to surgery, immediately postoperatively, and at euthanasia. Blood samples were taken and analyzed for white blood cell count (WBC) preoperatively, at day 3 and 15 postoperatively, and at euthanasia. Euthanasia was performed on day 20 postoperatively using an intravenous overdose (6000 mg/sheep) of pentobarbital (Esconarkon^®^, Streuli Pharma AG, Uznach, Switzerland).

### 4.8. Sample Harvest, Ex Vivo Radiology, Quantitative Bacteriology, and Histology

After euthanasia, anteroposterior contact radiographs were made of the screw area (cabinet x-ray systems, Faxitron, Tucson, AZ, USA). The 3 most distal screws were removed from each sheep post-mortem using a digital torque measuring tool (Mecmesin, Advanced Force and torque indicator) under sterile conditions. The removed screws were subsequently placed in separate glass vials containing 7 mL of PBS. These were sonicated for 10 min (Bandelin Ultrasonic water bath, model RK 510 H operating at 640 W) and serial 10-fold dilutions were performed using PBS. The samples were plated on blood agar (BA) and trypticase soy agar (TSA) and incubated for 20–24 h at 37 °C. The colonies were then counted at the dilution yielding the densest, yet countable, colonies. The exposed screw holes were scraped with a curette, and scrapings were collected in lactated ringers and centrifuged, and the pellets were suspended in 4% paraformaldehyde (PFA) + 2.5% glutaraldehyde (McDowell’s fixative) for later electron microscopy. Slabs of approximately 2.5 cm were cut around the screw holes with a butcher saw and stored in McDowell’s fixative. After fixation, samples were decalcified in EDTA, embedded in paraffin, and processed for transmission electron microscopy (TEM), as previously described [7,23]. For each sheep, the most proximal screw was left in situ, and a slab of approximately 2.5 cm was cut around it. The slab was fixed in 70% methanol and embedded in methyl methacrylate. Slides were cut and stained with Giemsa and Eosin (G&E).

## 5. Conclusions

This study described the development of a clinically relevant model of an ODRI caused by MRSA in four sheep. The results of these case reports support prospective studies comparing vancomycin monotherapy to vancomycin and HBCS co-administration to treat screws contaminated with 10^3^ CFU of MRSA.

## Data Availability

Dataset available on request from the authors.

## Abbreviations

The following abbreviations are used in this manuscript:

HBCS	Hydroxybisphosphonate conjugated sitafloxacin

## References

[1] Urish K.L., Cassat J.E. (2020). *Staphylococcus aureus* Osteomyelitis: Bone, Bugs, and Surgery. Infect. Immun..

[2] Masters E.A., Ricciardi B.F., Bentley K.L.M., Moriarty T.F., Schwarz E.M., Muthukrishnan G. (2022). Skeletal infections: Microbial pathogenesis, immunity and clinical management. Nat. Rev. Microbiol..

[3] Moriarty T.F., Metsemakers W.J., Morgenstern M., Hofstee M.I., Vallejo Diaz A., Cassat J.E., Wildemann B., Depypere M., Schwarz E.M., Richards R.G. (2022). Fracture-related infection. Nat. Rev. Dis. Primers.

[4] Metsemakers W.J., Smeets B., Nijs S., Hoekstra H. (2017). Infection after fracture fixation of the tibia: Analysis of healthcare utilization and related costs. Injury.

[5] Bezstarosti H., Van Lieshout E.M.M., Voskamp L.W., Kortram K., Obremskey W., McNally M.A., Metsemakers W.J., Verhofstad M.H.J. (2019). Insights into treatment and outcome of fracture-related infection: A systematic literature review. Arch. Orthop. Trauma Surg..

[6] de Mesy Bentley K.L., Trombetta R., Nishitani K., Bello-Irizarry S.N., Ninomiya M., Zhang L., Chung H.L., McGrath J.L., Daiss J.L., Awad H.A. (2017). Evidence of *Staphylococcus aureus* Deformation, Proliferation, and Migration in Canaliculi of Live Cortical Bone in Murine Models of Osteomyelitis. J. Bone Miner. Res..

[7] de Mesy Bentley K.L., MacDonald A., Schwarz E.M., Oh I. (2018). Chronic Osteomyelitis with *Staphylococcus aureus* Deformation in Submicron Canaliculi of Osteocytes: A Case Report. JBJS Case Connect..

[8] Masters E.A., de Mesy Bentley K.L., Gill A.L., Hao S.P., Galloway C.A., Salminen A.T., Guy D.R., McGrath J.L., Awad H.A., Gill S.R. (2020). Identification of Penicillin Binding Protein 4 (PBP4) as a critical factor for *Staphylococcus aureus* bone invasion during osteomyelitis in mice. PLoS Pathog..

[9] Adjei-Sowah E., Peng Y., Weeks J., Jonason J.H., de Mesy Bentley K.L., Masters E., Morita Y., Muthukrishnan G., Cherian P., Hu X.E. (2021). Development of Bisphosphonate-Conjugated Antibiotics to Overcome Pharmacodynamic Limitations of Local Therapy: Initial Results with Carbamate Linked Sitafloxacin and Tedizolid. Antibiotics.

[10] Sedghizadeh P.P., Sun S., Junka A.F., Richard E., Sadrerafi K., Mahabady S., Bakhshalian N., Tjokro N., Bartoszewicz M., Oleksy M. (2017). Design, Synthesis, and Antimicrobial Evaluation of a Novel Bone-Targeting Bisphosphonate-Ciprofloxacin Conjugate for the Treatment of Osteomyelitis Biofilms. J. Med. Chem..

[11] Sedghizadeh P.P., Cherian P., Roshandel S., Tjokro N., Chen C., Junka A.F., Hu E., Neighbors J., Pawlak J., Russell R.G.G. (2023). Real-Time Impedance-Based Monitoring of the Growth and Inhibition of Osteomyelitis Biofilm Pathogen *Staphylococcus aureus* Treated with Novel Bisphosphonate-Fluoroquinolone Antimicrobial Conjugates. Int. J. Mol. Sci..

[12] Weinstein R.S., Roberson P.K., Manolagas S.C. (2009). Giant osteoclast formation and long-term oral bisphosphonate therapy. N. Engl. J. Med..

[13] Rogers M.J. (2004). From molds and macrophages to mevalonate: A decade of progress in understanding the molecular mode of action of bisphosphonates. Calcif. Tissue Int..

[14] Trombetta R.P., Dunman P.M., Schwarz E.M., Kates S.L., Awad H.A. (2018). A High-Throughput Screening Approach To Repurpose FDA-Approved Drugs for Bactericidal Applications against *Staphylococcus aureus* Small-Colony Variants. mSphere.

[15] Ren Y., Xue T., Rainbolt J., Bentley K.L.M., Galloway C.A., Liu Y., Cherian P., Neighbors J., Hofstee M.I., Ebetino F.H. (2022). Efficacy of Bisphosphonate-Conjugated Sitafloxacin in a Murine Model of *S. aureus* Osteomyelitis: Evidence of “Target & Release” Kinetics and Killing of Bacteria Within Canaliculi. Front. Cell. Infect. Microbiol..

[16] Trombetta R.P., Ninomiya M.J., El-Atawneh I.M., Knapp E.K., de Mesy Bentley K.L., Dunman P.M., Schwarz E.M., Kates S.L., Awad H.A. (2019). Calcium Phosphate Spacers for the Local Delivery of Sitafloxacin and Rifampin to Treat Orthopedic Infections: Efficacy and Proof of Concept in a Mouse Model of Single-Stage Revision of Device-Associated Osteomyelitis. Pharmaceutics.

[17] Kuhn E.M.A., Sominsky L.A., Chitto M., Schwarz E.M., Moriarty T.F. (2024). Antibacterial Mechanisms and Clinical Impact of Sitafloxacin. Pharmaceuticals.

[18] Ren Y., Weeks J., Xue T., Rainbolt J., de Mesy Bentley K.L., Shu Y., Liu Y., Masters E., Cherian P., McKenna C.E. (2023). Evidence of bisphosphonate-conjugated sitafloxacin eradication of established methicillin-resistant *S. aureus* infection with osseointegration in murine models of implant-associated osteomyelitis. Bone Res..

[19] Salamaga B., Kong L., Pasquina-Lemonche L., Lafage L., von Und Zur Muhlen M., Gibson J.F., Grybchuk D., Tooke A.K., Panchal V., Culp E.J. (2021). Demonstration of the role of cell wall homeostasis in *Staphylococcus aureus* growth and the action of bactericidal antibiotics. Proc. Natl. Acad. Sci. USA.

[20] Sutton J.A.F., Carnell O.T., Lafage L., Gray J., Biboy J., Gibson J.F., Pollitt E.J.G., Tazoll S.C., Turnbull W., Hajdamowicz N.H. (2021). *Staphylococcus aureus* cell wall structure and dynamics during host-pathogen interaction. PLoS Pathog..

[21] Xie C., Ren Y., Weeks J., Rainbolt J., Kenney H.M., Xue T., Allen F., Shu Y., Tay A.J.H., Lekkala S. (2024). Longitudinal intravital imaging of the bone marrow for analysis of the race for the surface in a murine osteomyelitis model. J. Orthop. Res..

[22] Nishitani K., Sutipornpalangkul W., de Mesy Bentley K.L., Varrone J.J., Bello-Irizarry S.N., Ito H., Matsuda S., Kates S.L., Daiss J.L., Schwarz E.M. (2015). Quantifying the natural history of biofilm formation in vivo during the establishment of chronic implant-associated *Staphylococcus aureus* osteomyelitis in mice to identify critical pathogen and host factors. J. Orthop. Res..

[23] de Mesy Bentley K.L., Galloway C.A., Muthukrishnan G., Echternacht S.R., Masters E.A., Zeiter S., Schwarz E.M., Leckenby J.I. (2021). Emerging electron microscopy and 3D methodologies to interrogate *Staphylococcus aureus* osteomyelitis in murine models. J. Orthop. Res..

